# Antimicrobial resistance and virulence factors in *Escherichia coli *from swedish dairy calves

**DOI:** 10.1186/1751-0147-54-2

**Published:** 2012-01-26

**Authors:** Kerstin de Verdier, Ann Nyman, Christina Greko, Björn Bengtsson

**Affiliations:** 1 Department of Animal Health and Antimicrobial Strategies, National Veterinary Institute, 751 89 Uppsala, Sweden

## Abstract

**Background:**

In Sweden, knowledge about the role of enteropathogenic *Escherichia coli *in neonatal calf diarrhea and the occurrence of antimicrobial resistance in *E. coli *from young calves is largely unknown. This has therapeutic concern and such knowledge is also required for prudent use of antimicrobials.

**Methods:**

In a case control study *Esherichia coli *isolated from faecal samples from dairy calves were phenotyped by biochemical fingerprinting and analyzed for virulence genes by PCR. Antimicrobial susceptibility was tested by determination of minimum inhibitory concentration (MIC). Farm management data were collected and Fisher's exact test and univariable and multivariable logistic regression analysis were performed.

**Results:**

Of 95 *E. coli *tested for antimicrobial susceptibility 61% were resistant to one or more substances and 28% were multi-resistant. The virulence gene F5 (K99) was not found in any isolate. In total, 21 out of 40 of the investigated virulence genes were not detected or rarely detected. The virulence genes *espP, irp*, and *fyuA *were more common in resistant *E. coli *than in fully susceptible isolates (*P *< 0.05). The virulence gene *terZ *was associated with calf diarrhea (*P *≤ 0.01).

The participating 85 herds had a median herd size of 80 lactating cows. Herds with calf diarrhea problems were larger (> 55 cows; P < 0.001), had higher calf mortality (P ≤ 0.01) and calf group feeders were more in use (*P *< 0.05), compared to herds without calf diarrhea problems.

There was no association between calf diarrhea and diversity of enteric *E. coli*.

**Conclusions:**

Antimicrobial resistance was common in *E. coli *from pre-weaned dairy calves, occurring particularly in calves from herds experiencing calf diarrhea problems. The results indicate that more factors than use of antimicrobials influence the epidemiology of resistant *E. coli*.

Enteropathogenic *E. coli *seems to be an uncommon cause of neonatal calf diarrhea in Swedish dairy herds. In practice, calf diarrhea should be regarded holistically in a context of infectious agents, calf immunity, management practices etc. We therefore advice against routine antimicrobial treatment and recommend that bacteriological cultures, followed by testing for antimicrobial susceptibility and for virulence factors, are used to guide decisions on such treatment.

## Background

Neonatal calf diarrhea (NCD) is a major disease worldwide when calves are reared intensively, and constitute substantial cost in terms of calf mortality, opportunity costs for labor and capital, veterinary costs and loss in calf value. The term NCD generally refers to a disease complex characterized by acute, undifferentiated diarrhea in young calves. It is a multifactorial disease where, besides the causative pathogenic agent, calf age, management and environmental factors, may influence the clinical outcome [1].

A large number of infectious agents have been incriminated as causes of NCD, including *Salmonella *and bovine viral diarrhea virus (BVDV). Commonly reported causative pathogens are rotavirus, coronavirus, *Cryptosporidium *spp and enteropathogenic *Escherichia coli *(*E. coli*) [2-5]. Enteric colibacillosis is manifested primarily by varying degrees of diarrhoea and dehydration and the outcome may be fatal. The major virulence factors of enteropathogenic strains of *E. coli *in NCD are the F5 (K99) adhesion antigen and also the heat-stable enterotoxin (ST) [6]. Under Swedish conditions, *E. coli *is of practitioner's concern from a therapeutic perspective. In most cases of NCD in Swedish dairy calves antimicrobial therapy is not indicated since most enteric *E. coli *strains from calves are non pathogenic [7]. However, for treatment of enteritis caused by enteropathogenic *E. coli*, or of *E. coli *bacteraemia secondary to enteritis, antimicrobials given orally or systemically are recommended [8]. Knowledge of the susceptibility is necessary for elaboration of guidelines on prudent use of antimicrobials.

Although it is a potential pathogen, *E. coli *is a normal inhabitant of the gastrointestinal tract of most warm blooded animals and one of the most common representatives of the aerobic gram-negative microbiota. Such commensal bacteria can be reservoirs for transferable resistance genes and thereby reflect the selective pressure from use of antimicrobials in a population [9,10]. Enteric *E .coli *from healthy animals is therefore recommended as indicators for prevalence of resistance in animal populations [11] and used in several monitoring programs.

In Sweden, the role of enteropathogenic *E. coli *in NCD is only fragmentarily known [7,12-14]. Likewise, the knowledge on occurrence of antimicrobial resistance in *E. coli *from young calves is scarce. Therefore, the aim of this study was to investigate among *E. coli *from preweaned dairy calves the occurrence of virulence genes and of antimicrobial resistance and their association. The aim was also to evaluate the association between the occurrence of NCD and antimicrobial resistance, virulence genes and potential risk factors.

## Methods

### Herds

Field veterinary practitioners all over Sweden were invited to submit samples from dairy herds by their own choice, for a case/control study. One inclusion criterion was BVDV-free dairy herds; i e. participation in the national BVD control program and declared free from BVDV. Other inclusion criteria were > 29 cows, and that case and control herds should have experienced > 6 and < 3 diarrhoeic calves respectively, out of the 20 most recently born.

### Samples

Samples were collected from March 2004 to June 2005. Faecal samples were collected as rectal swabs. According to instructions, case/control pairs of calves should be sampled, comprising one healthy (i. e. no signs of diarrhoea) calf in each control herd (CD-) and one calf with acute diarrhoea in each case herd (CD+). Case/control pairs should comprise calves of equal age, and all the sampled calves should be between one day and four weeks old. Calves should not have been treated with antimicrobials within two weeks prior to sampling. The samples were submitted by ordinary mail to the Swedish National Veterinary Institute (SVA), and arrived there the day after sampling.

### Questionnaire

Data were collected from the farmers by a questionnaire. Questions were asked about the herd (number of cows, number of unweaned calves); calf age, breed and gender; calf management (time for cow-calf separation ("< 24 h after birth", "1-3 days after birth" and ">3 days after birth"), age at weaning, feeding (use of calf group feeder) and rearing systems); routines for antimicrobial treatment (use of dihydrostreptomycin^1 ^(DHS) orally for treatment of diarrhoea ("never", "sometimes", or "often") and use of other antimicrobials orally for treatment of diarrhoea ("never", "sometimes", or "often"); calf mortality (of the 20 most recently born calves); information on calf health i.e. general appearance of sampled calves ("not affected", "slightly to moderately affected", and "severely affected"), number of calves with diarrhoea in the herd (of the 20 most recently born calves), diarrhea consistency ("soft", "watery" and "bloody"), diarrhea duration ("1 day", "2 days" and "3 days"), respiratory signs ("yes" and "no").

### Bacteriological culture

The samples were streaked on horse blood agar (5% v/v) and on MacConkey agar and incubated at 37°C for 18 h. From each sample, 25 colonies that morphologically corresponded to *E. coli *were sub-cultured on horse blood agar [15]. Isolates were confirmed as *E. coli *by testing for production of tryptophanase (indole) and β-glucuronidase (p-nitrophenyl- β-D-glucopyanosiduronic acid, PGUA). Only lactose-positive isolates with typical morphology and positive reaction in both tests were selected for further analysis.

All 25 selected *E. coli *isolates from each sample were phenotyped by biochemical fingerprinting [16]. Briefly, the method is based on evaluation of the kinetics of biochemical reactions and performed in micro titer plates containing 11 different dehydrated reagents (The Phene Plate System; PhP-system, Biosys AB, Stockholm, Sweden). The isolates were hence clustered into different PhP types, describing the diversity of *E. coli *in the sample. Based on the biochemical fingerprinting, one isolate representing the dominant phenotype in each sample was selected for testing of antimicrobial susceptibility and virulence genes.

### Analysis of antimicrobial susceptibility

Antimicrobial susceptibility was tested by determination of minimum inhibitory concentration (MIC) using a microdilution method. Testing was performed according to recommendations by CLSI (formerly NCCLS) (NCCLS, 2002) using VetMIC™ panels (National Veterinary Institute, Uppsala, Sweden) and cat ion adjusted Mueller-Hinton broth (Becton Dickinson, Cockeysville, USA). Antimicrobials and range of concentrations tested are given in Table 1. The quality control strain, *E. coli *ATCC 25922, tested in parallel with each batch of isolates, was on all occasions within acceptable ranges. Apart from florfenicol the tested antimicrobials are, or have been, licensed for use in cattle in Sweden.

**Table 1:** Resistance (percent, 95% CI in brackets) and distribution (percent) of MICs for ***Escherichia coli ***(n = 95).

Antimicrobial	**Range tested **(mg/L)	**Cut-off value **(mg/L)	Resistance	**Minimum Inhibitory Concentration **(mg/L)																	
				≤0.03	0.06	0.12	0.25	0.5	1	2	4	8	16	32	64	128	256	512	1024	2048	>2048
Ampicillin	0.25-32	>8	27.4 (18.7-37.5)				0	0	11.6	48.4	12.6	0	**0**	**1.1**	**26.3**						
Ceftiofur	0.12-16	>1	0.0 (0.0-3.8)			0	38.9	53.7	7.4	**0**	**0**	**0**	**0**								
Chloramphenicol	1-128	>16	5.3 (1.7-11.9)						1.1	9.5	74.7	9.5	0	**0**	**0**	**0**	**5.3**				
Enrofloxacin	0.03-4	>0.12	13.7 (7.5-22.3)	18.9	61.1	6.3	**3.2**	**6.3**	**2.1**	**1.1**	**0**	**1.1**									
Florfenicol	4-32	>16	0.0 (0.0-3.8)								66.3	30.5	3.2	**0**							
Gentamicin	0.5-64	>2	0.0 (0.0-3.8)					25.3	68.4	6.3	**0**	**0**	**0**	**0**	**0**						
Nalidixic acid	1-128	>16	13.7 (7.5-22.3)						2.1	31.6	51.6	1.1	0	**1.1**	**3.2**	**4.2**	**5.3**				
Neomycin	2-16	>8	5.3 (1.7-11.9)							87.4	5.3	2.1	**0**	**5.3**							
Streptomycin	2-256	>16	44.2 (34.0-54.8)							1.1	16.8	32.6	5.3	**2.1**	**14.7**	**8.4**	**10.5**	**8.4**			
Sulphonamide	16-2048	>256	31.6 (22.4-41.9)										47.4	17.9	3.2	0	0	**0**	**1.1**	**1.1**	**29.5**
Tetracycline	0.5-64	>8	31.6 (22.4-41.9)						37.9	27.4	2.1	1.1	**0**	**1.1**	**0**	**30.5**					
Trimethoprim	0.25-32	>2	5.3 (1.7-11.9)				37.9	41.1	14.7	1.1	**1.1**	**0**	**0**	**0**	**4.2**						

Range of concentrations tested and cut-off values for resistance are indicated. MICs equal to or lower than the lowest concentration tested are given as the lowest tested concentration. MICs above the range of concentrations tested are given as the concentration closest to the range. MICs above the cut-off value for resistance are given in bold lettering.

Isolates were classified as susceptible or resistant based on epidemiological cut-off values issued by European Committee on Antimicrobial Susceptibility Testing (EUCAST; http://www.escmid.org). These cut-off values classify an isolate as resistant to an antimicrobial when its MIC is distinctly higher than those of inherently susceptible, "wild type", strains of the bacterial species. For sulphametoxazole no cut-off value is available from EUCAST, therefore a provisional value was set according to the principles for epidemiological cut-off values. Cut off-values used are given in Table 1.

### Analysis of virulence genes

Isolates were analyzed for 10 different virulence genes (*F4, F5, F6, F18, F41, STa, STb, LT, EAST, VT2e*) by PCR at SVA (inhouse PCR, Swedish National Veterinary Institute). A subset of isolates were tested for an additional 30 virulence genes (*VT1, VT2, eae, beta, gamma, alpha, kappa, epsilon, fimA, fimB, fimC, fimH, fimE, fliC, etpD, bfpA, hlyA, espA, espB, espP, katP, terA, terC, terW, terE, terZ, ehly1, irp, fyuA, astA*) by PCR at the Veterinary Laboratories Agency (VLA), Weybridge, UK (RM La Ragione personal communication). The subset of isolates, selected with attention to differences in calf age and geographical location, were submitted in cryo-tubes with agar by ordinary mail to VLA.

### Statistical methods

This study was performed as a case-control study, where the calf was the study unit and the dependent variable was disease status, classified as case (calves diagnosed with diarrhea; CD+) or control (calf diagnosed not to have diarrhea; CD-). Unconditional associations between the dependent variable and each of the independent variables were first screened using Fisher's exact test and univariable logistic regression analysis. Variables with a *P*-value ≤ 0.20, provided that there was no co linearity (r < 0.70) between variables, were then considered for further analysis. Collinearity between variables was assessed pair-wise by calculation of Spearman rank correlations.

Moreover, associations between antimicrobial resistance and presence of virulence genes in isolates of *E. coli *were investigated using the same analyses as in the case-control part of the study.

Continuous variables, not linearly related to the outcome, were categorized using the quartiles as cut-points, or by using biologically important, or recommended cut-points. Categories of categorical variables with too few observations were amalgamated when biological, or logical, new categories were possible to make. In other cases such categorical variables were not used in the analysis. Variables with many missing values (> 20% missing observations) were not used in the multivariable analysis.

A multivariable model was constructed using manual stepwise backward logistic-regression analysis, where variables not significant in the model were re-entered whenever a new variable became significant, or a variable was removed. Potential confounders and intervening factors were considered in every model. A variable was considered as a confounder if the point estimates of the coefficients in a model changed > 20% with the potential confounder present. In the final model a variable with a *P*-value ≤0.05 was considered statistically significant and retained in the model. Biologically plausible interactions between the main effects were tested in the final model. Herd was not included as a random factor due to too few observations per herd. However, the "cluster" command in Stata was used making the standard errors allow for intragroup correlation.

The fit of the models was evaluated with the Hosmer-Lemeshow goodness-of-fit test with the data partitioned into 10 deciles. The statistical analyses were done using Stata Software (StataCorp., 2010; Stata Statistical Software: Release 11.0; College Station, TX, USA: StataCorp LP.).

## Results

Samples were obtained from a total of 104 calves. From these, samples from 95 calves, representing 85 dairy herds, were used for laboratory and statistical analyses. Exclusion of samples was due to failure of isolating *E. coli *(no growth or overgrowth by *Proteus *spp). Of the 95 calves, 56 were diagnosed as having diarrhea.

All information about the herds was collected by the questionnaire used at the farm visit. However, all questions were not always fully answered for all herds, hence there are a varying number of missing values for the variables included in this study. The participating 85 herds were located in all geographical regions of Sweden with significant cattle population and had a median herd size of 80 lactating cows (50% central range (CR): 40 - 140 cows). The calves were of both the Swedish red and white breed (n = 51) and of the Swedish Holstein breed (n = 42). Forty-four of the calves were heifer calves and 49 were bull calves. The calves were separated from their dam < 24 h after birth (n = 48), 1-3 days after birth (n = 23) or after > 3 days (n = 21). Calf group feeder was used in 36 of the herds.

On average 1.7 calf (50% CR: 0 - 3 calves) and 8.8 calves (50% CR: 5 - 10 calves) out of the 20 latest born calves had had diarrhoea in herds with CD- calves and CD+, respectively. In herds with CD- calves 0.5 calf of the 20 latest born calves (50% CR: 0 - 1) had died, and in herds with CD+ calves 1.4 calves of the 20 latest born calves (50% CR: 0 - 2) had died.

Twenty-three of the CD+ calves were diagnosed with diarrhoea in the first 10 days after birth, 13 calves were diagnosed 11-21 days after birth, and 19 calves 22-135 days after birth. In the same periods 11, 18 and 10 of the CD- calves were sampled. Of the diarrhoeic calves 70% had soft diarrhoea, 21% watery diarrhoea and 9% had blood in the faeces. Twenty-four percent of the calves were sampled one day after the onset of diarrhoea, 33% two days after, 40% three days after, and 3% 6-14 days after. The general appearance was affected in 56% of the CD+ calves (43.6% "slightly to moderately"; 12.7% "severely"). None of the CD- calves had an affected general appearance. Only 9% of all participating calves had respiratory symptoms, and these were all CD+ calves.

In 32% of the herds dihydrostreptomycin tablets (DHS) were occasionally used to treat diarrhoea, and in 44% of the herds other antimicrobials were used occasionally or often to treat diarrhoea.

*E. coli *was isolated in samples from all participating calves. One isolate from each calf (n = 95) was analysed for antimicrobial susceptibility and 94 isolates for occurrence of 10 virulence genes. Moreover, 48 isolates (13 from calves with diarrhoea) were analysed for 30 additional virulence factors.

### Univariable analysis

The result from the univariable analysis are presented in Table 2 and 3 for variables associated with being a CD+ or CD- calf and for variables associated with antimicrobial resistance in *E. coli *isolates (P < 0.20).

**Table 2:** Results from the univariable logistic regression analysis of variables significantly (P ≤ 0

Variable	Level	Healthy calves	Diarrheic calves	P-value
Herd factors				
Breed	*0: Swedish red and white*	18	33	
	*1: Swedish Holstein*	21	21	
	*2: Other*	0	1	0.15*
Age at weaning	*0: 6-8 weeks of age*	22	20	
	*1: 9-11 weeks of age*	7	15	
	*2: 12-16 weeks of age*	7	13	0.20*
Proportion of dead calves/20 born calves	*0: 0%*	24	19	
	*1: 5%*	11	12	
	*2: ≥ 10%*	4	18	0.01
Proportion of calves having diarrhea/20 born calves	*0: < 5%*	16	1	
	*1: 5-20%*	17	7	
	*2:>20%*	6	42	< 0.001
Age at sampling	*0: 0-11 days*	11	23	
	*1: 12-21 days*	18	13	
	*2: ≥ 22 days*	10	19	0.07*
Herd size (no of cows)	*0: < 55*	21	10	
	*1: 55-120*	14	19	
	*2: ≥ 121*	4	25	< 0.001*
Milk calf feeder?	*0: No*	28	28	
	*1: Yes*	10	26	0.03*
Usage of other antibiotics to treat diarrhea	*0: Never*	30	20	
	*1: Sometimes/Often*	7	33	< 0.001
				
Antimicrobial resistance				
Resistant to ampicillin	*0: No*	34	35	
	*1: Yes*	7	19	0.05*
Resistant to streptomycin	*0: No*	27	26	
	*1: Yes*	14	28	0.08*
Resistant to tetracycline	*0: No*	36	29	
	*1: Yes*	5	25	< 0.01*
Resistant to sulphonamide	*0: No*	34	31	
	*1: Yes*	7	23	< 0.01*
				
Multiresistant (resistant to at least three antibiotics (Am, Sm, Tc and/or Su))	*0: No*	37	37	
	*1: Yes*	4	19	< 0.01
				
Presence of virulence genes				
Presence of terZ	*0: No*	24	1	
	*1: Yes*	11	10	< 0.01
Presence of astA	*0: No*	28	8	
	*1: Yes*	5	5	0.09
Presence of EAST	*0: No*	33	37	
	*1: Yes*	6	18	0.06*

* variables eligible for entering the multivariable analysis

**Table 3:** Results from the univariable logistic regression analysis of variables significantly (P ≤ 0.20) associated with *Escherichia coli *isolates (n = 95) with or without antimicrobial resistance (from 95 healthy or diarrheic calves from 85 Swedish dairy herds).

Variable	Level	*E. coli *with antimicrobial resistance	*E. coli *without antimicrobial resistance	P-value
Herd factors				
Age at sampling	*0: 0-11 days*	24	10	
	*1: 12-21 days*	13	16	
	*2: ≥ 22 days*	19	10	0.09*
Any respiratory syndromes	*0: No*	50	33	
	*1: Yes*	7	1	0.10
Herd size (no of cows)	*0: < 55*	14	17	
	*1: 55-120*	20	13	
	*2: ≥ 121*	21	6	0.04*
Time for separation of cow and calf	*0: < 24 h after calving*	33	14	
	*1: 1-3 days after calving*	12	10	
	*2: > 3 days after calving*	9	12	0.09*
Usage of DHS tablets to treat diarrhea	*0: Never*	40	21	
	*1: Sometimes/Often*	15	15	0.20*
				
Presence of virulence genes				
Presence of espP	*0: No*	11	19	
	*1: Yes*	15	3	0.002*
Presence of irp	*0: No*	12	19	
	*1: Yes*	14	3	0.003*
Presence of fyuA	*0: No*	13	18	
	*1: Yes*	13	4	0.02*

* variables eligible for entering the multivariable analysis

#### Herd factors

There was no significant association between breed, gender, time to separation from dam, age of diagnosis and being a CD+ or CD- calf (*P *> 0.05). However, both breed and age of diagnosis had a P-value < 0.20 and were considered for the multivariable regression analysis. There were a significant higher proportion of calves having diarrhoea, and a higher proportion of calves that had died, in herds with CD+ calves than herds with CD- calves (P ≤ 0.01). CD+ calves came from larger herds (> 55 cows) than CD- calves (*P *< 0.001), and from herds where it was more common with calf group feeder (*P *< 0.05). The use of other antimicrobials than DHS to treat diarrhoea was more common in herds with CD+ calves (*P *< 0.001).

#### Diversity

The median diversity of the *E. coli *isolates was 0.50 (50% CR: 0.23 - 0.72). There was no significant difference in diversity between samples from CD+ and CD- calves. Also, there was no difference in diversity between samples where the dominating PhP type was resistant to one or more antimicrobials and where the dominating type was not resistant.

#### Virulence genes

Of the virulence genes, *fimA, fimB, fimC, fimE, fimH, fliC*, and *terA *were found in ≥ 90% of the isolates, but there were no significant difference in findings between isolates from CD+ or CD- calves. The virulence genes *terZ, terW, espP, irp, fyuA, EAST *and *astA *were found in 46%, 42%, 37.5%, 35%, 35%, 25% and 22% of the isolates, respectively. The gene *terZ *was significantly more often found in *E. coli *from CD+ calves than CD- calves (*P *≤ 0.01).

The virulence genes *bfpA, espB, ehly1, F4, F5, F6, F18*, and *LT *were not found in any isolate. Moreover, in ≥ 87.5% of the isolates *eae, espA, etpD, F41, hlyA, katP, STa, STb, terE, terC, VT1, VT2*, and *VT2e *were absent.

#### Antimicrobial resistance

Of the 95 *E. coli *tested for antimicrobial susceptibility 61% were resistant to one or more substances. Distribution of MIC values and percentages of *E. coli *resistant to the antimicrobials tested are presented in Table 1. Twenty-seven isolates (28%) were multiresistant, i.e. resistant to three or more antimicrobials. Of these, 19 isolates had resistance to streptomycin, sulphonamide and tetracycline in the phenotype, usually in combination with other resistance traits. Significantly more isolates from CD+ calves than from CD- calves were resistant to ampicillin, tetracycline or sulphonamide (*P *≤ 0.05). Also multiresistance, including resistance to ampicillin, streptomycin, tetracycline and sulphonamide, was more common (*P *≤ 0.01) among isolates from CD+ calves. There was no significant association between antimicrobial resistance of the isolates and herd routines for use of DHS, or other antimicrobials, to treat diarrhoea.

#### Virulence genes and associations with antimicrobial resistance

The virulence genes *espP, irp*, and *fyuA *were more common in *E. coli *resistant to one or more antimicrobials than in fully susceptible isolates (*P *< 0.05).

### Multivariable analysis

#### Variables associated with being a CD+ or CD- calf

A total of 72 variables were screened in the univariable logistic regression analysis of variables associated with being a CD+ or CD- calf. Of these 72 variables 10 (with a *P *≤ 0.20 and considered being potential risk factors) were eligible for entering the multivariable analysis (Table 2). However, there was a high correlation (r ≥ 0.70) between herd size and milk calf feeder, as well as for presence of resistance against streptomycin and sulphonamide. Hence, of these variables, the variable with the lowest *P*-value in the univariable analysis were selected to be eligible for the multivariable analysis. In the final multivariable analysis of factors associated with being a CD+ or a CD- calf three variables remained with a *P *≤ 0.05 (Table 4). There was an increased risk of being a CD+ calf for a calf diagnosed at age < 11 or ≥ 22 days old compared to calves diagnosed at 12-21 days old. Moreover, there was a higher risk that a calf in a larger herd (≥ 120 cows) was a CD+ calf than a calf in a smaller herd (< 120 cows), and that CD+ calves more often had *E. coli *isolates that were resistant to tetracycline.

**Table 4:** Final multivariable logistic regression analysis of variables significantly (***P ***≤ 0

Variable	*β*	S.E.(*β*)	OR^a^	95% CI (OR^a^)	*P*-value
Intercept	1.24	0.57	-	-	-
Herd size					
*0: ≥ 121*	Ref^b^	-	-	-	-
*1: 55-120*	-1.52	0.74	0.22	0.05; 0.93	0.04
*2: < 55*	-2.28	0.70	0.10	0.03; 0.40	0.001
Resistant to tetracycline?					
*0:No*	Ref^b^	-	-	-	-
*1:Yes*	1.66	0.64	5.25	1.48; 18.6	0.01

^a^OR = Odds ratio

^b^Ref = reference category

The final model showed reasonably good fit; the Hosmer-Lemeshow χ^2 ^(8 d.f.) was 4.7 (*P *= 0.79).

#### Variables associated with antimicrobial resistance in E. coli isolates

A total of 58 variables were screened in the univariable logistic regression analysis of variables associated with antimicrobial resistance in *E. coli *isolates. Of these 58 variables 7 (with a *P *≤ 0.20, and considered being potential risk factors) were eligible for entering the multivariable analysis (Table 3). However, there was a high correlation (r ≥ 0.70) between presence of *irp *and *fyua*, and the variable with the lowest P-value in the univariable analysis was selected to be eligible for the multivariable analysis. It was not possible to make any final multivariable model since the only variable remaining with a *P*-value ≤ 0.05 in the model was herd size.

## Discussion

This study shows that antimicrobial resistance is widespread in enteric *E. coli *from healthy as well as from diarrheic preweaned dairy calves. Streptomycin, sulphonamide, tetracycline or ampicillin were the most prevalent resistance traits and isolates resistant to all these antimicrobials were common. The findings are in agreement with studies in preweaned dairy calves from other countries [17-22]. Also quite similar results were obtained from Swedish calves sampled at post-mortem [23]. In such calves a high occurrence of resistance can be anticipated since a large proportion of the animals are probably treated with antimicrobials. An equally high prevalence of resistant *E. coli *in the untreated calves of the present study is therefore remarkable.

As discussed by Call et al. [24] the epidemiology of resistant *E. coli *in calves is multifactorial, complex and e.g. influenced by co-selection due to linkage of resistance genes. But widespread resistance is fundamentally a consequence of historical and current use of antimicrobials and associations between use of antimicrobials and resistance in enteric *E. coli *of calves have been documented [19,20,22,25-28]. However, in the present study resistance is not a direct sequel to antimicrobial use since no calf was treated prior to sampling. Antimicrobial use in calves is still not uncommon in Sweden and Ortman & Svensson [29] showed that in dairy herds about one third of diarrhoeic calves (1-90 days) were treated with trimethoprim/sulfa, enrofloxacin or other antimicrobials. Also in the present study NCD was treated with antimicrobials in several herds but there was no statistical association between such routines and resistance. Although the total use of antimicrobials in the herds is unknown, the absence of association indicates that the high prevalence of resistance is not solely an effect of a direct selection pressure by use of antimicrobials to the calves.

Importance of other aspects than antimicrobial use on prevalence of resistant *E. coli *in preweaned calves is indicated by several studies [17,20-22,26,28,30] and was recently reviewed by Call et al. [24]. One proposed factor is a linkage between resistance genes and genes conferring selective advantage to colonize the intestinal lumen of calves. Walk et al. [28] hypothesized that, regardless of use of antimicrobials, tetracycline resistance in *E. coli *is co-selected in calves by an unknown "beneficial mutation". Likewise, Khachatryan et al. [21,30] showed that *E. coli *with the resistance phenotype streptomycin - sulfonamide - tetracycline have a selective advantage to colonize the intestine of calves given a dietary milk supplement also in absence of antimicrobials. Notably, in the present study about one third of the resistant isolates and two thirds of the multiresistant isolates had streptomycin - sulfonamide - tetracycline in their phenotype.

A selective advantage of resistant strains due to dietary differences is in agreement with the age related occurrence of resistant *E. coli *documented elsewhere [18-20,22,28,31]. The importance of age is evident also on comparison of data from the present study to previous data from older cattle in Sweden, in which resistant *E. coli *are rare [23].

Another factor of possible importance for resistance in preweaned calves is feeding milk from cows treated with antimicrobials or feeding colostrum from cows treated in the dry period [17,20,32,33]. It has been proposed that antimicrobial residues of such milk could select for resistance in the enteric flora of calves. Although there are few studies to support the assumptions, the risk of a "hidden" selection pressure in dairy calves warrants further studies of the issue.

In the present study, resistance was more common in *E. coli *from calves with diarrhea than in isolates from healthy calves. Also multivariate analysis showed that resistance to tetracycline in *E. coli *was associated with diarrhea in calves. A higher occurrence of resistance in *E. coli *in calves from herds experiencing problems with NCD was shown also by Gunn et al. [34]. A plausible reason for this is that antimicrobials are used more often in herds with a high disease incidence, as indicated in the present study by the more common routine of treating NCD with antimicrobials in CD+ herds than in CD- herds. However, a higher prevalence of resistant *E. coli *in calves with diarrhoea could also be due to linkage between virulence genes and resistance genes as proposed by Martinez & Baquero [35]. Such linkage was demonstrated in *E. coli *from pigs with diarrhoea [36,37] and could imply co-selection of virulence genes by use of antimicrobials and conversely maintenance of resistance in populations of pathogenic bacteria as proposed by Boerlin et al. [36]. In the present study phenotypic resistance to one or more antimicrobials was associated with presence of the virulence genes *espP, irp *or *fyuA *in *E. coli*. However, there was no association to single antimicrobials or resistance phenotypes. Moreover, none of these virulence factors were associated with diarrhea and the findings should be interpreted with caution. Likewise, Suojala et al. [38] found an association between the virulence factor *iucD *and resistance to streptomycin, ampicillin, sulphametoxazole and trimethoprim in *E. coli *from dairy cows with mastitis, but they found no association between the virulence factor and patogenicity.

Attention has been drawn by veterinary practitioners to the possibility that *E. coli *is a more prevalent cause of NCD than demonstrated in previous Swedish studies, restricted to *E. coli *with virulence factor F5. This suggestion is supported by international studies showing a higher prevalence of *E. coli *F5 than in the Swedish studies [39-42]. This virulence gene was not found in any isolate in this study and 21 out of 40 of the investigated genes were not detected or rarely detected. Although the number of isolates tested was small, this contradicts a common occurrence of enteropathogenic *E. coli *in NCD. A possible exception is *E. coli *with the virulence gene *terZ *which was associated with diarrhea. However, further studies are needed to clarify clinical relevance of these virulence genes in NCD.

The etiology of diarrhea in this study was not clarified, since other infectious agents than *E. coli *were not searched for. The impact of *Salmonella *and BVDV, however, was most likely none, since the prevalence of *Salmonella *spp is low in Swedish cattle herds [43] and all the participating herds were declared BVD-free.

In the present study there was no association between diversity of enteric *E. coli *and calf diarrhea. A decrease in the homogeneity of the faecal coliform flora has been shown in suckling pigs with diarrhea [44]. Therefore, biochemical fingerprinting was performed to increase the probability of selecting isolates of the pathogenic strain and thereby the probability of detecting virulence genes. The results in the present study agree with the findings of Acha et al. [39] and could indicate that in calves, there is no predominance of single clones even in diarrhea caused by enteropathogenic *E. coli*. Still, in the present study it more likely indicates that diarrhea caused by *E. coli *was uncommon since there, with one exception, was no association between occurrence of virulence genes and diarrhea.

In most studies of antimicrobial susceptibility in intestinal bacteria, isolates are randomly selected from a culture of intestinal content. But in the present study, isolates of the most common phenotype in each sample was selected for testing. This did not influence the overall prevalence of resistance in the group of calves sampled, but in individual calves it occasionally had a profound effect on the outcome of the susceptibility test. Accordingly, to guide the choice of antimicrobial therapy it can be misleading to test *E. coli *with undefined clinical relevance. Moreover, this emphasizes the advantage of identifying pathogenic isolates for susceptibility testing, possibly by detection of virulence factors.

Sampled calves deviated slightly from the given inclusion criteria, i e 0-3 (CD-) and 5-10 (CD+) of the 20 most recently born calves had been diarrheic, as compared to 0-2 (CD- criterion) and > 6 (CD+ criterion). Other sampling errors were a mismatch in age at sampling between CD+ and CD- calves, and an extended age interval for CD+ and CD- calves (1-135 days, mean 21.9) compared to the criteria (four weeks). Nevertheless, there were clear differences in clinical signs between CD- and CD+ calves and CD- and CD+ herds regarding mortality, occurrence of diarrhea, general appearance and respiratory signs. One contributing reason for this could be that CD+ herds were larger than CD- herds. Larger herds were more likely to have calf group feeder in the present study, and there might be a larger number of neonatal calves in the herd during the same time period which increases the risk for infectious enteric diseases. The association between diarrhea in calves and large herd size agrees with the findings in a recent Swedish study on calf morbidity and mortality in herds with different size (C Sandgren personal communication). The calf mortality in this study was higher than previously reported in Swedish herds [45] which also could reflect a negative impact of large herd size on calf health. Calf rearing practices have changed concurrently with herd size.

## Conclusions

Antimicrobial resistance was common in *E. coli *from preweaned dairy calves and particularly in calves from herds experiencing problems with neonatal diarrhea. Resistance could not be associated to use of antimicrobials, implying that other factors also influence the epidemiology of resistant *E. coli*. Isolates with virulence genes were as common in calves with as without clinical signs.

In practice, neonatal calf diarrhea should be seen holistically in a context of infectious agents, calf immunity and management practices. This study indicates that enteropathogenic *E. coli *is an uncommon cause of NCD in Swedish dairy herds. We therefore advice against routine antimicrobial treatment and recommend that bacteriological cultures, followed by testing for antimicrobial susceptibility and for virulence factors, are used to guide decisions on such treatment

## Endnotes

^1^Dihydrostreptomycin vet, tablets, Boehringer Ingelheim, Vetmedica, Malmö, Sweden

## Competing interests

The authors declare that they have no competing interests.

## Authors' contributions

KdV and BB designed and coordinated the study and drafted the manuscript. AN performed the statistical analysis and helped to draft the manuscript. CG participated in study design and helped to draft the manuscript. All authors read and approved the final manuscript.
